# Sinus node dysfunction in a young patient with Hodgkin lymphoma: a case report

**DOI:** 10.1093/ehjcr/ytaf360

**Published:** 2025-08-07

**Authors:** Gabriela-Elena Marascu, Madalina Schmidt, Ruxandra Vidlescu, Mihai Manciu, Eliza Cinteza, Radu-Gabriel Vatasescu

**Affiliations:** Faculty of Medicine, Carol Davila University of Medicine and Pharmacy, Eroii Sanitari Boulevard, No 8, District 5, Bucharest 050474, Romania; Department of Cardiology, Clinical Emergency Hospital of Bucharest, Calea Floreasca, No 8, District 1, Bucharest 014461, Romania; Department of Oncology, Marie Curie Emergency Children’s Hospital, Constantin Brancoveanu Boulevard, No 20, District 4, Bucharest 041451, Romania; Faculty of Medicine, Carol Davila University of Medicine and Pharmacy, Eroii Sanitari Boulevard, No 8, District 5, Bucharest 050474, Romania; Department of Oncology, Marie Curie Emergency Children’s Hospital, Constantin Brancoveanu Boulevard, No 20, District 4, Bucharest 041451, Romania; Department of Cardiology, Marie Curie Emergency Children’s Hospital, Constantin Brancoveanu Boulevard, No 20, District 4, Bucharest 041451, Romania; Faculty of Medicine, Carol Davila University of Medicine and Pharmacy, Eroii Sanitari Boulevard, No 8, District 5, Bucharest 050474, Romania; Department of Cardiology, Marie Curie Emergency Children’s Hospital, Constantin Brancoveanu Boulevard, No 20, District 4, Bucharest 041451, Romania; Faculty of Medicine, Carol Davila University of Medicine and Pharmacy, Eroii Sanitari Boulevard, No 8, District 5, Bucharest 050474, Romania; Department of Cardiology, Clinical Emergency Hospital of Bucharest, Calea Floreasca, No 8, District 1, Bucharest 014461, Romania

**Keywords:** Hodgkin lymphoma, Sinus node dysfunction, Arrhythmia, Cardiac nuclear imaging, Case report

## Abstract

**Background:**

Sinus node dysfunction is uncommon among bradyarrhythmias in patients with lymphomas, and it has never been reported in those with Hodgkin lymphoma (HL). We present a case of a young male diagnosed with HL who exhibited asymptomatic sinus node dysfunction.

**Case summary:**

A 17-year-old male was diagnosed with stage IV A nodular sclerosis classic type HL. The electrocardiogram showed intermittent sinus arrest with a junctional rhythm. There was no evidence of structural changes in the right atrium (RA) walls during the initial transthoracic echocardiography evaluation. Computed tomography staging revealed multiple mediastinal adenopathies that infiltrated the cardiac level, extending into the RA and interatrial septum. The positron emission tomography scan showed metabolically active adenopathies above the diaphragm and in the upper abdomen, with nuclear uptake primarily in the RA. The patient’s conduction and rhythm disorders improved during chemotherapy, highlighting the cardiac involvement linked to the underlying disease.

**Discussion:**

To our knowledge, this is the first documented case of HL presenting with sinus node dysfunction as an early sign of cardiac involvement.

Learning points

- Bradyarrhythmia related to Hodgkin lymphoma can be reversed with timely chemotherapy, without the need for temporary or permanent pacing.
- We should consider using multimodality imaging techniques like positron emission tomography because diffuse infiltration can happen.
- The 24-h Holter electrocardiogram monitoring is useful for identifying subclinical cardiac damage and assessing the response to chemotherapy.

## Introduction

Lymphoma is one of the most frequent malignancies with the potential for cardiac metastasis. Hodgkin lymphoma (HL) in children is most frequently seen during adolescence. There are numerous histological types, with nodular sclerosing HL being the most prevalent.^1^ While the staging process is comparable for both Hodgkin and non-Hodgkin lymphomas, the presence and the extent of cardiac damage in HL remains uncertain. Cardiovascular involvement typically manifests as focal masses. Diffuse invasion generally cannot be detected by simple imaging tools. Conduction abnormalities as a direct cardiac involvement of initial phases of HL were never been reported in the literature, and are often observed in non-HL.^2^ Conduction and rhythm disturbances can include bradycardia, atrioventricular block, atrial fibrillation (AF) or flutter, and more rarely sinus node dysfunction. It could result from lymphoma infiltration into the heart (as is often the case for non-HL) or can be a side effect of chemotherapy (in the overwhelming majority of HL before metastatic phase). The use of ^18^F fluorodeoxyglucose (FDG) positron emission tomography (PET) for functional imaging has become the standard practice for the first assessment and treatment of HL due to the high FDG-PET avidity of this tumour.^1^ The early initiation of chemotherapy can resolve bradyarrhythmia in cardiac lymphomas, without the need for invasive strategies.

We report a 17-year-old male diagnosed with HL who presented with asymptomatic sinus node dysfunction, including sinus arrest, junctional rhythm, and AF.

## Summary figure

**Figure:**
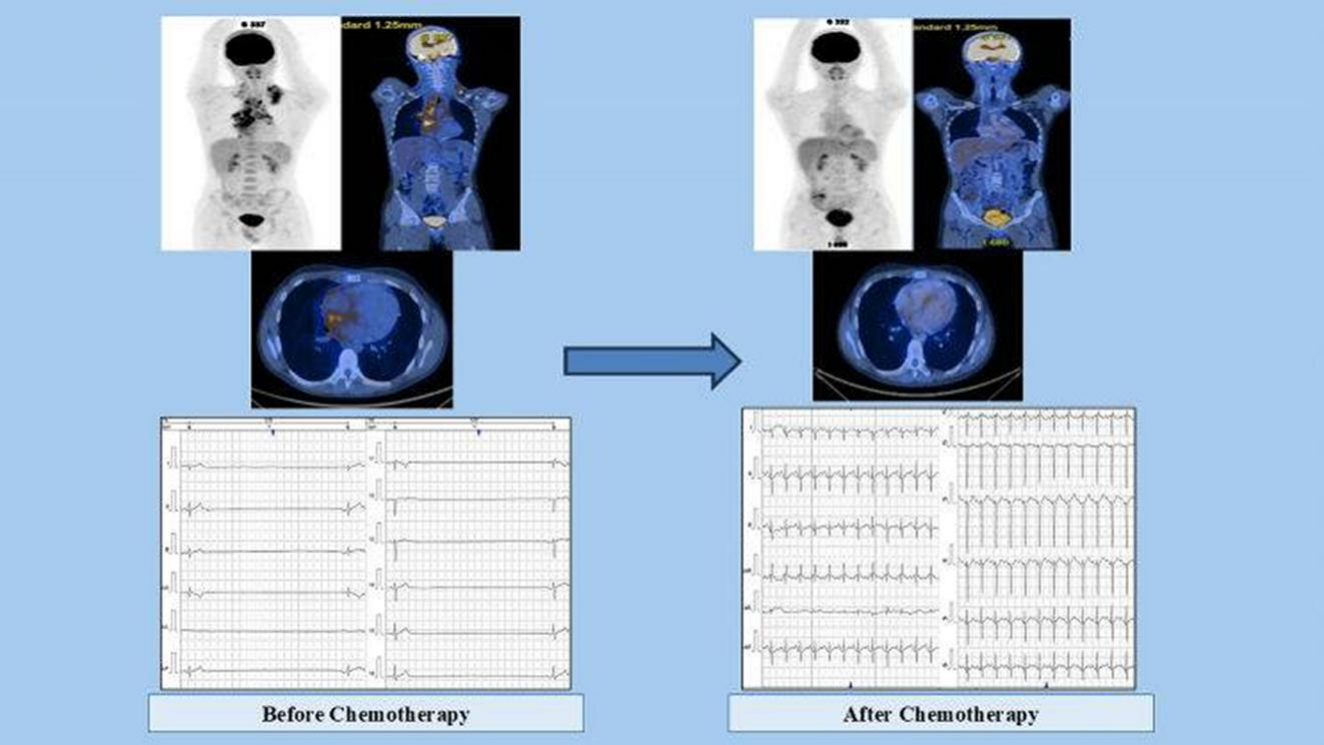


## Case presentation

A 17-year-old male was diagnosed with stage IV A nodular sclerosis classic type HL. The first manifestation of the disease was the appearance of a left laterocervical and left supraclavicular adenopathy, with progressive growth, accompanied by intermittent episodes of generalized itching. The histopathological examination of the left laterocervical ganglion established the diagnosis. The laboratory tests revealed a negative serology for the Ebstein–Barr virus and Cytomegalovirus.

**Figure 1:**
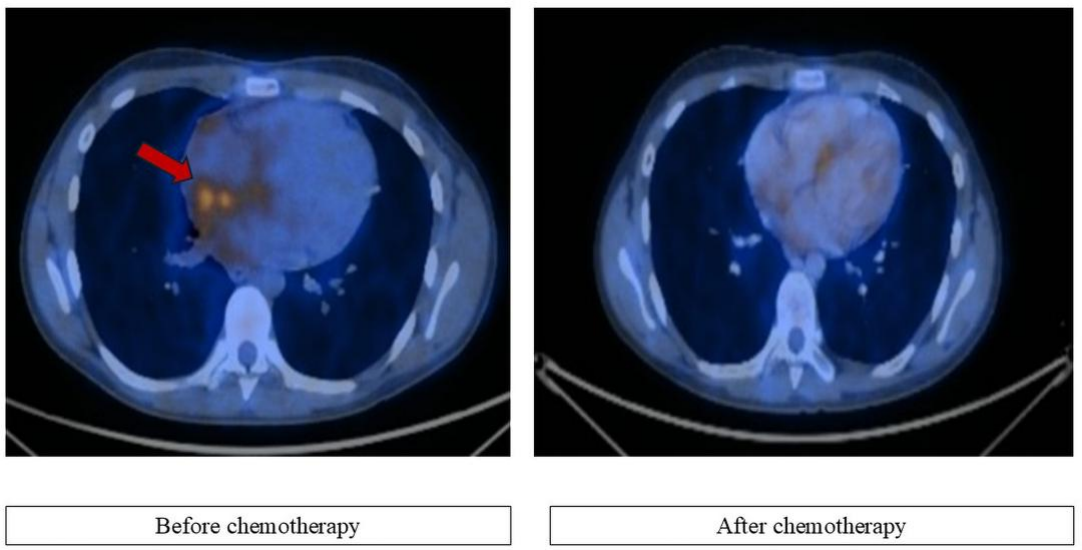
Cardiac nuclear imaging investigation; PET-CT scan before and after initiation of chemotherapy; red arrow indicates extensive FDG uptake predominantly at RA level, with a clear decrease after chemotherapy.

Computed tomography (CT) examination performed during staging revealed the presence of left laterocervical adenopathy, bilateral supraclavicular adenopathy, predominantly on the left side, and multiple mediastinal adenopathies. The mediastinal adenopathies merged and infiltrated at the bilateral hilar level, extending downward into the intracardiac region, specifically in the superior wall of the right atrium (RA) and interatrial septum. The patient also underwent PET-CT whole-body FDG scan, which revealed metabolically active adenopathies above the diaphragm and in the upper abdomen, and nuclear uptake in the heart, mainly in the RA (*Figure 1*).

**Figure 2:**
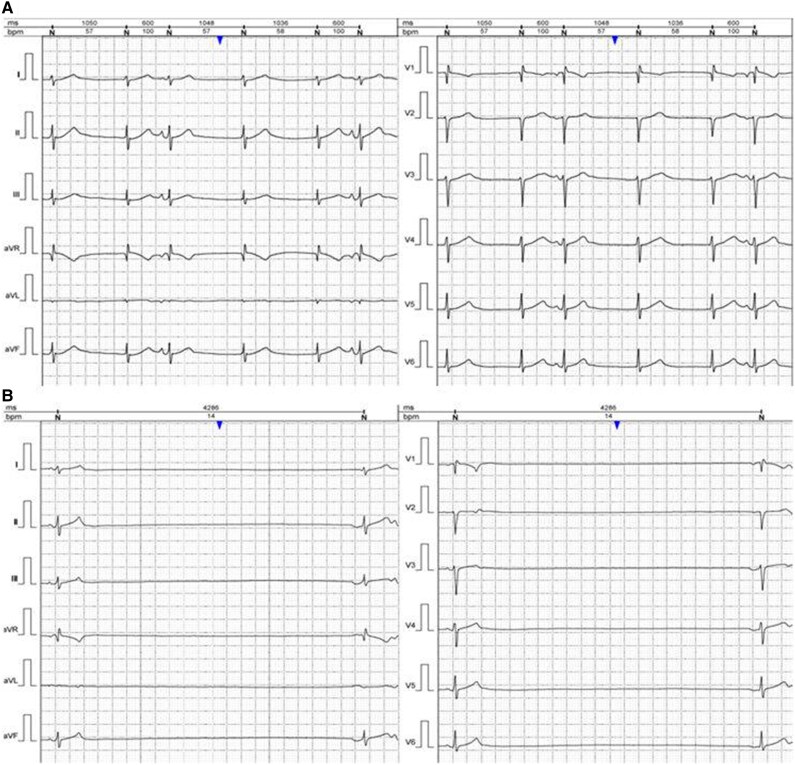
The 24-h Holter-ECG monitoring; junctional rhythm, heart rate ∼60 b.p.m. with supraventricular extrasystoles (*A*), sinus pause above 4286 ms (*B*).

**Figure 3:**
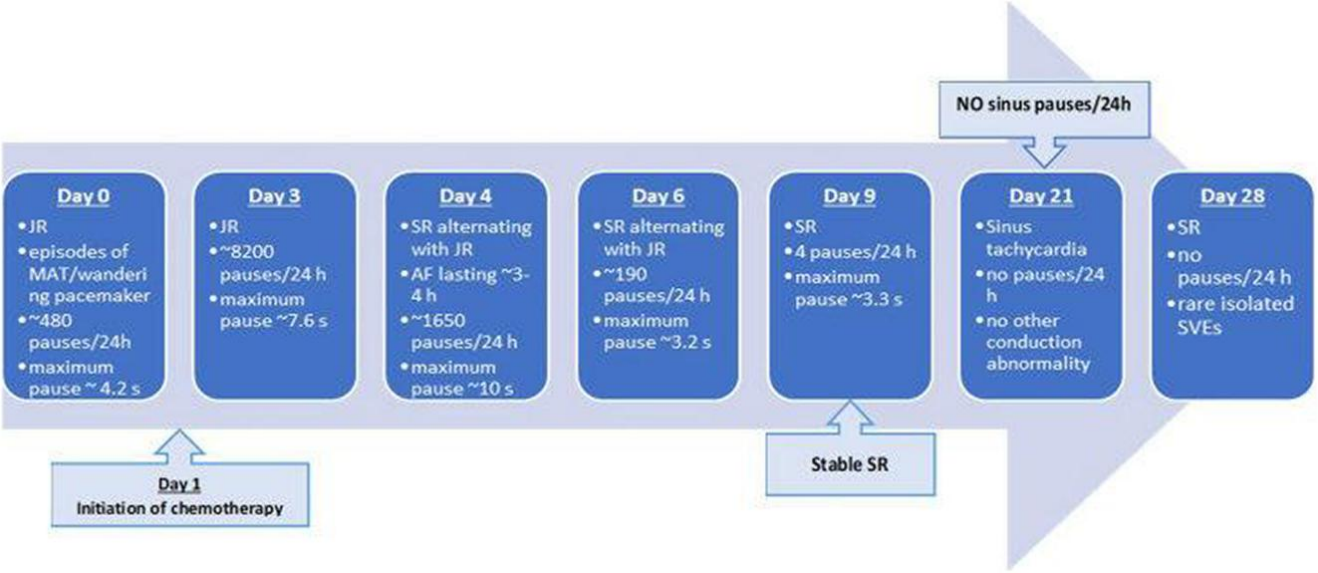
Holter-ECG monitoring in dynamic; during chemotherapy, sinus rhythm is restored, and sinus pauses cease within 21 days after treatment starts. JR, junctional escape rhythm; MAT, multifocal atrial tachycardia; SR, sinus rhythm; AF, atrial fibrillation; SVEs, supraventricular extrasystoles.

He was hospitalized for re-evaluation and initiation of the chemotherapy protocol. The electrocardiogram (ECG) showed a junctional rhythm with multiple supraventricular extrasystoles and intermittent sinus arrest. There was no evidence of structural heart disease during the clinical examination and transthoracic echocardiography (TTE). The first 24-h Holter-ECG monitoring showed a background escape junctional rhythm, atrial bigeminy, short episodes of multifocal atrial tachycardia, and intermittent sinus arrest. The maximum pause was ∼4.2 s, with around 480 pauses per 24 h lasting more than 3 s, predominantly occurring at night (*Figure 2*). The patient showed no symptoms related to the rhythm disturbances observed on the surface ECG or Holter-ECG recordings. The next day, treatment with methylprednisolone sodium succinate was initiated, with doses increasing over time. Two days later, the patient began the OEPA 1 chemotherapy protocol (methylprednisolone, vincristine, etoposide, and doxorubicin). The patient underwent 24-h Holter-ECG monitoring in dynamics, with a maximum sinus pause of ∼10 s (*Figure 3*). During the following period, the patient experienced an episode of AF on Holter monitoring. Beginning on Day 21 of chemotherapy, the patient no longer showed sinus pauses on the 24-h Holter-ECG recordings (*Figure 3*).

**Figure 4:**
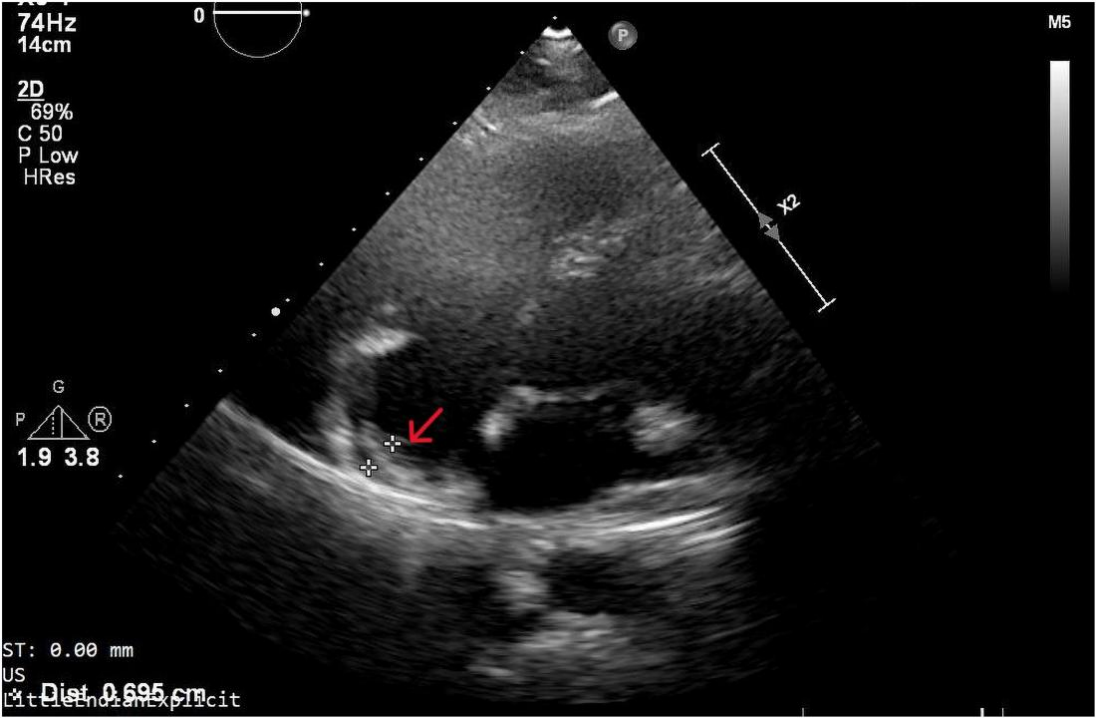
Transthoracic echocardiography; right atrium with hyperechogenic walls, and a mass of ∼7 mm on the superior wall extending to the interatrial septum, indicated by the red arrow.

**Figure 5:**
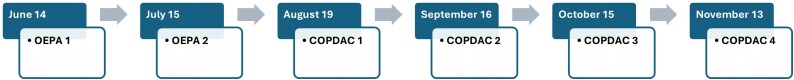
The chemotherapy protocol administered; chronological evolution of the chemotherapy treatments followed by the patient. OEPA, methylprednisolone, vincristine, etoposide, and doxorubicin; COPDAC, methylprednisolone, vincristine, dacarbazine, and cyclophosphamide.

Next month, the patient performed a full body CT, revealing an infiltrative mediastinal mass expansion. The extension was observed at the posteroinferior wall of the RA and the interatrial septum, as well as at the level of the left common carotid artery and superior vena cava. The TTE evaluation showed RA with hyperechogenic walls, thickened superiorly and extending towards the left atrium (*Figure 4* and Supplementary material online, *Video S1*). On July 15, the second cycle of the OEPA protocol began (*Figure 5*).

The PET-CT whole-body ^18^F-FDG scan was repeated in August. Compared to the investigation carried out in June, it indicates dimensional and metabolic regression of the supradiaphragmatic adenopathy, without abdominal adenopathy with increased metabolic activity, and accumulation in the RA (*Figure 1*). On echocardiography, the disappearance of the structural changes at the level of the RA was noted. The 24-h Holter monitoring revealed sinus rhythm, with bigeminy supraventricular extrasystoles and without pauses or another rhythm or conduction anomalies. On 19 August, COPDAC 1 cure (methylprednisolone, vincristine, dacarbazine, and cyclophosphamide) was initiated and well tolerated, followed by COPDAC 2, 3, and 4 without significant adverse effects (*Figure 5*).

## Discussions

Cardiac lymphoma is a rare disease classified as primary or secondary, with secondary being the most prevalent type.^3–5^ Cardiac involvement can develop through haematogenous dissemination, retrograde lymphatic spread, or direct extension from mediastinal lymphoma.^3,6^ Three growth patterns—heart wall infiltration, isolated pericardial effusion (more frequently), and intracaval masses—can be seen in lymphomas.^4^ Although the staging method is similar for both types of lymphomas, the cardiac damage in HL is still unclear.

Typically, lymphomas infiltrate more than one cardiac structure while sparing cardiac valves. The most common intracardiac sites are the RA and right ventricle.^4^ When a RA mass is present, other possible diagnoses include vegetations, thrombus forms, and cardiac or non-cardiac malignancies.^5^ In our patient, TTE initially did not reveal any structural changes, as advanced imaging techniques are needed to confirm RA involvement. Combining conventional CT with metabolic imaging using PET was crucial for diagnosis, staging, and assessing therapeutic response.

According to the type of cancer, the oncological treatment, the patient’s features, and risk factors, there are differences in the prevalence of arrhythmias in oncological patients.^7^ Chemotherapy or the cancer itself can cause atrial and more rare, ventricular arrhythmias. Cancer patients may experience an imbalance in the autonomic nervous system, primarily due to ongoing pain, chronic inflammation and oedema, and other forms of stress associated with cancer treatment.^7,8^

Patients with cancer have a higher incidence of AF than the general population.^9^ It has been suggested that AF occurs in the first 3 months to 1 year after a cancer diagnosis.^9^ There is a strong association between the onset of AF and haematological cancers (lymphoma, leukaemia, multiple myeloma), intrathoracic tumours, and central nervous system cancers.^9^ The pro-inflammatory state and the increased inflammatory response to chemotherapy, radiation, and surgical procedures are some of the pathological conditions that lead to structural and electrical atrial remodelling.^9^ Chemotherapeutics such as anthracyclines, tyrosine kinase inhibitors, fluoropyrimidines, or melphalan are linked to the highest incidence rate of developing AF.^7–9^ The most recent ESC Guidelines for the diagnosis and treatment of AF should be followed when handling AF in a patient with cancer.^10^

Most bradyarrhythmia in cancer patients are symptomless and can easily go undetected.^7^ Presenting symptoms in cancer patients may include paraneoplastic symptoms such as fever, weakness, fatigue, or symptoms related to thrombo-embolic or haemodynamic issues or arrhythmias.^5^ Therefore, in younger patients, these may be underdiagnosed because their symptoms resemble those of the underlying oncological disease. Pre-syncope, syncope, fatigue, and dizziness include several possible symptoms. It can happen as a side effect of cancer treatment, as a result of vagus nerve involvement in the tumours, or as a result of cancer infiltrating the cardiac conduction system.^7^ In our case, the patient was asymptomatic, and the Holter-ECG monitoring was valuable in assessing the severity of the bradyarrhythmias.

Among types of bradyarrhythmia, sinus node dysfunction is rare in patients with lymphomas, and have never been documented for HL. Until now, only one case of supraventricular arrhythmias in a patient with HL has been stated.^2^ Sanders *et al*.^11^ reported a case of a primary cardiac lymphoma with T-cell morphology presenting as sinus node dysfunction with progressive atrial myopathy and paroxysmal atrial flutter. Similarly to our case, their patient denied syncope or dizziness, and the conduction disorder was highlighted by telemetry. Also, macroscopic evidence of a cardiac tumour was not initially evident, but unlike our case, pericardial effusion was present. They concluded that small, localized tissue changes associated with tumour infiltration led to gradual disturbance of atrial electrical activity.^11^

The patient’s conduction and rhythm disorders improved during chemotherapy, as shown by Holter-ECG monitoring, supporting the cardiac involvement related to the underlying disease. Martz *et al*.^3^ found that lymphomas associated with noncritical conduction disease may have more opportunities to avoid temporary or permanent pacemaker implantation due to their sensitivity to chemotherapy and radiotherapy. Hirakawa *et al*. reported the effective management of symptomatic sick sinus syndrome in a patient with cardiac non-HL using only chemotherapy. They proposed that arrhythmias related to lymphoma’s infiltration in the cardiac conduction system can be reversed with timely chemotherapy.^12^

## Conclusions

Electrocardiogram monitoring in our patient suggested cardiac lymphoma extension, highlighting the need for ongoing suspicion even if TTE is normal. Multimodality imaging should be considered since diffuse infiltration may occur.

This case illustrates that arrhythmias linked to HL can be reversible with prompt chemotherapy, with a favourable effect on the prognosis of these patients. Thus, in patients with HL, 24-h Holter-ECG monitoring can be useful both to detect subclinical cardiac damage and to assess treatment response.

To our knowledge, this is the first reported case of HL presenting with sinus node dysfunction as an early manifestation of cardiac involvement.

## Supplementary Material

ytaf360_Supplementary_Data

## Data Availability

Data used in this paper is available upon request to the corresponding author.

## Lead author biography

I graduated from Carol Davila University of Medicine and Pharmacy in 2021. I am currently a resident doctor in Cardiology at the Clinical Emergency Hospital of Bucharest and a PhD student at Carol Davila University of Medicine and Pharmacy. My primary research interests focus on arrhythmias and advanced cardiac imaging techniques.
